# Supplementary material for: Loess deposits in the low latitudes of East Asia reveal the ~20-kyr precipitation cycle
Source: Nat Commun. 2024 Feb 3;15:1023. doi: 10.1038/s41467-024-45379-9 (PMC10838313; doi:10.1038/s41467-024-45379-9)
Supplement: Supplementary file 1 — Supplementary Information [file 41467_2024_45379_MOESM1_ESM.pdf]

Supplementary information for

Loess deposits in the low latitudes of East Asia reveal the ~20-kyr precipitation cycle

Xusheng Li[1], Yuwen Zhou[1], Zhiyong Han[1]*, Xiaokang Yuan[1], Shuangwen Yi[1], Yuqiang Zeng[1], Lisha Qin[1], Ming Lu[1] & Huayu Lu[1]

[1] School of Geography and Ocean Science, Nanjing University, Nanjing 210023, China

* Corresponding author. Email: zyhan@nju.edu.cn

1. Geographical settings

The Madang profile (29°59′29″ N, 116°39′44″ E) is located in Madang Town, Pengze County, Jiangxi Province, southern China (Supplementary Fig. 1). It is approximately 850 km from the Chinese Loess Plateau (CLP) in the northwest and 340 km from the Xiashu profile in the lower Yangtze River in the northeast. The Hukou–Tongling section of the lower Yangtze River, located between Dabie Mountain and the mountains of southern Anhui, forms a southwest–northeast oriented valley. This valley is about 200 km long and 60 km wide (Supplementary Fig. 1a). Within this region, Pengze County is located on the south bank of the Yangtze River, with the terrain gradually rising from northwest to southeast. The northwestern part of Pengze County consists of an alluvial plain along the river, with an average elevation of approximately 20 m. Sand hills are distributed in some areas along the river. In the central area, there are hills and basins with little topographic variation. Loess has accumulated in the piedmont and in the basins, and lakes have formed in the depressions of the basins. Moving towards the southeast area, the landscape is characterized by low and middle mountains and numerous gullies and river valleys. Eluvial deposits are widespread in this region (Supplementary Fig. 1b).

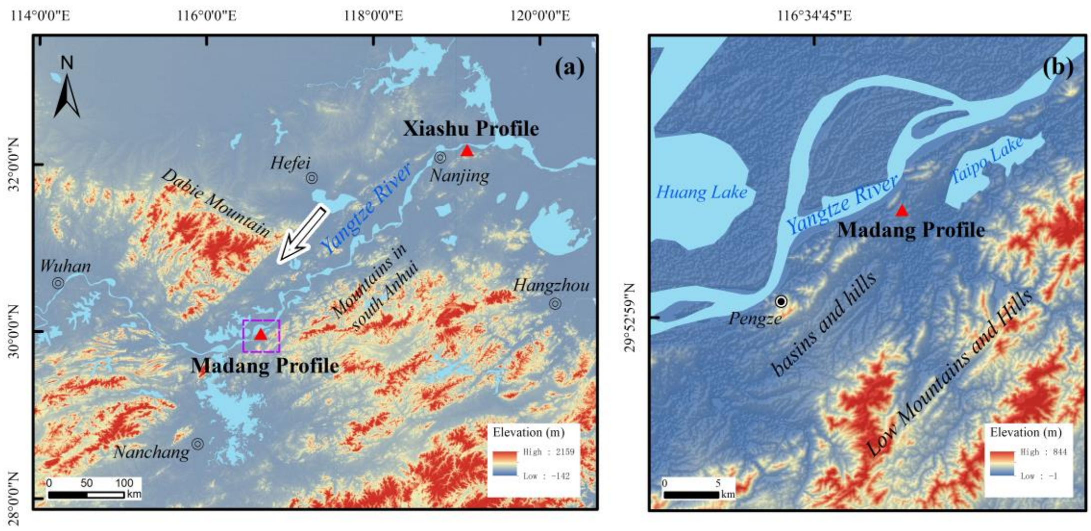

Supplementary Fig. 1. Terrain in the lower reaches of the Yangtze River (a) and the adjacent Madang profile (b). The arrow indicates the prevailing wind direction. The purple rectangle in (**a**) indicates the area of (**b**). A digital elevation model (SRTM V4)[1] used in the figure is available from the CGIAR-CSI SRTM 90m Database (http://srtm.csi.cgiar.org).

The intermittently distributed hills along the Yangtze River in Pengze County are about 100-300 m high and surround a basin about 5 km wide and 20 km long to the southeast, and the Madang profile lies within this basin. To the northwest of the profile is Huang Mountain (about 100 m a.s.l.), which is about 2 km long and 0.5 km wide, and to the northeast of the profile is Beng Mountain (about 100 m a.s.l.), which is about 3 km long and 1.5 km wide, and the two mountains are about 0.8 km apart. The Madang profile is located southeast of Huang Mountain, about 0.3 km away, 2 km from Huang Mountain, and 1 km from the Yangtze River, with an elevation of about 30 m at the top of this profile. The upper part of the profile is 13.2 m thick, and is exposed in an excavation pit, and the lower part is a 2.7 m long core, with a total thickness of 15.9 m.

The region has a subtropical monsoon climate, influenced by the East Asian monsoon circulation and the subtropical high pressure zone in summer and the cold high pressure of Mongolia and Siberia in winter. Influenced by the Yangtze River valley, which extends to the northeast, the northeasterly wind prevails near the surface. The mean annual temperature is 14–17 °C, the mean annual precipitation is 1100–1500 mm, the mean annual sunshine duration is 1917 hours, and the annual solar radiation is 113 kcal/cm^2. The ratio of April to September precipitation to annual precipitation is 67%

(Supplementary Fig. 2).

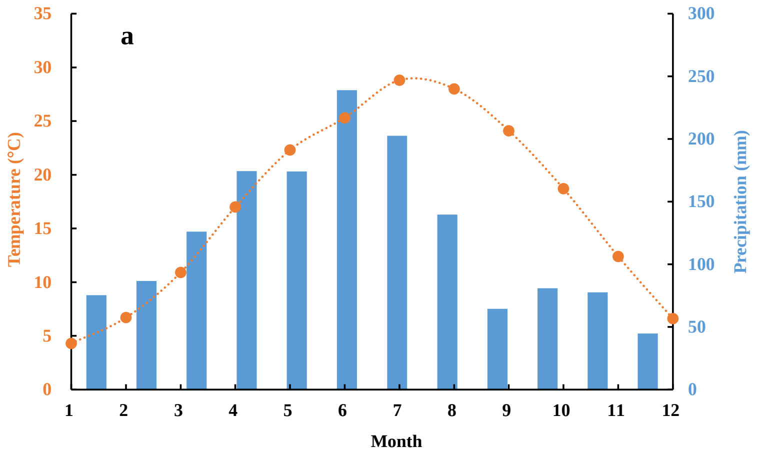

Supplementary Fig. 2. Monthly mean temperature and precipitation (1981-2000) at the Pengze meteorological station (http://data.cma.cn/).

2. Profile and sampling

The eolian sediments in the Madang profile are mainly characterised by their brownish-yellow colour, massive structure and vertical joints developed on outcrops, which are typical characteristics of loess. According to the sedimentary characteristics, the strata of the Madang profile can be divided into eight layers from top to bottom, which roughly correspond to the stratigraphic units L1, S1, L2, S2, L3, S3 and L4 of the CLP (Supplementary Fig. 3). The loess units are generally brownish yellow and the paleosol units are yellowish brown, forming a typical loess-paleosol sequence (Supplementary Table 1).

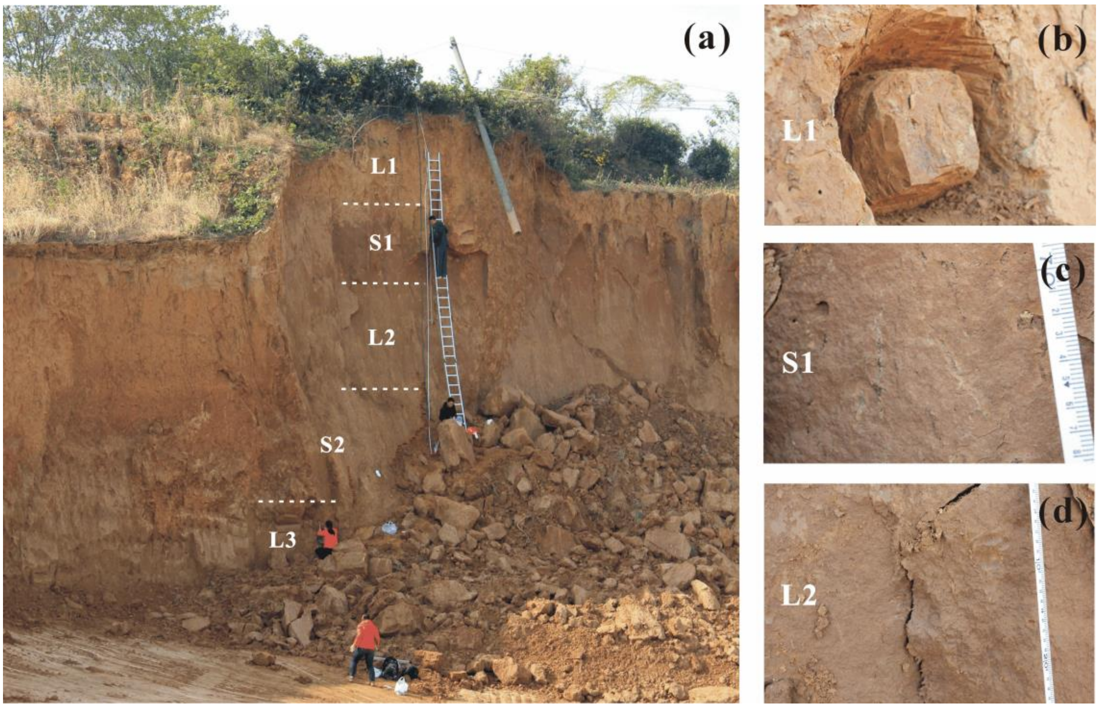

Supplementary Fig. 3. Outcrops of the Madang profile and lithology of the eolian sediments. (**a**) Stratigraphic subdivision following the scheme used in the Loess Plateau. The white dashed line represents the stratigraphic boundary. The visible strata thickness is 13.2 m; (**b-d**) The close-up photos of unit L1, S1 and L2, respectively.

Supplementary Table 1. Stratigraphic subdivision and lithological description of the Madang profile.

Depth (m)	Unit	Color	Description
0.0-2.5	L1	Grayish yellow	Silt, loose, massive, plant root
2.5-4.75	S1	Slight yellowish brown	Silt, hard and dense, massive, worm boring
4.8-7.6	L2	Slight brownish yellow	Silt, massive, unobvious Fe-Mn coating
7.65-8.95	S2	Slight yellowish brown	Silt, massive, unobvious Fe-Mn coating
9.0-10.1	L3	Slight brownish yellow	Silt, massive, few caesious mottle, unobvious Fe-Mn coating
10.15-11.1	S3	Slight brownish red	Silt, massive, obvious Fe-Mn coating in the middle part, few grey mottles in the middle and lower parts
11.15-15.9	L4	Slight brownish yellow	Silt, loose, massive, few grey mottle

The sampling interval is 5 cm for the upper part (<13.2 m) and 10 cm for the lower part (>13.2 m), with each sample being approximately 2 cm thick. A total of 291 bulk samples were collected for measurements of grain size, magnetic susceptibility (MS),

dithionite–citrate–bicarbonate (DCB) extractable iron (FeD), redness and hematite content. In addition, 6 samples were collected for optical stimulated luminescence (OSL) dating.

3. Methods

3.1. Grain size analysis

2 g of loess sample is placed in a beaker, and then 10 ml of 10% H_2O_2 is added and heated. Next, 10 ml of 10% HCl is added and the mixture is boiled. The beaker is then filled with distilled water and left to settle for 12 hours before the supernatant is decanted. Then 10 ml of 0.05 ml/L $(NaPO_3)_6$ dispersant is added, and the sample is shaken with an ultrasonic cleaner for 15 minutes. Finally, the sample is measured using a Malvern Panalytical Mastersizer2000 laser particle sizer. These measurements were carried out at the Laboratory of Surface Processes and Environment Evolution, Nanjing University.

3.2. Redness measurement

The redness of the sample was measured using a Konica–Minolta CM-700D spectrophotometer, which has a wavelength range of 400–700 nm and a test interval of 10 nm. The following steps were followed during the testing process: (1) The sample was naturally dried and ground to a particle size of less than 200 mesh. (2) The spectrophotometer was calibrated using a standard calibration white plate (CM-A177) with a standard deviation of spectral reflectance of less than 0.1% and a standard deviation of chromaticity (ΔE*ab) less than 0.04. (3) A suitable amount of sample was placed on a glass disc and flattened. (4) Three random measurements of the sample surface were taken with spectrophotometer to obtain the redness (a*) value of the sample. The redness measurements were conducted at the Institute of Geology, Chinese Academy of Geological Sciences.

3.3. Hematite measurement

The Hematite content is measured by diffuse reflectance spectroscopy (DRS)[2]. Before

conducting the DRS tests, the samples are pre-treated by the DCB dissolution method, which is carried out as follows: (1) A total of 21 samples of different depths are mixed and ground to a particle size of 200 mesh and then placed in a centrifuge cup. (2) The samples are heated to 75°C, then 45 ml of 0.3 N citrate and 5 ml of 1 N bicarbonate are added and mixed thoroughly. (3) Excess sodium dithionite is added to the mixture and stirred for 10 minutes. (4) The cup is centrifuged at 670.8 × g for 5 minutes. (5) Steps (2) to (4) are repeated. (6) 70 ml of distilled water is added to the sample for ultrasonic dispersion, and the cup is centrifuged at 2795 × g for 30 minutes. (7) The sample is reground to a particle size of 200 mesh to obtain the natural matrix.

Synthetic hematite and goethite standard minerals are incorporated into the natural matrix at various ratios to create a set of 26 standard samples. This includes one pure matrix sample, eight pure hematite samples, eight pure goethite samples, and nine mixed hematite and goethite samples (Supplementary Table 2). The hematite standard mineral used is a pure red nanoscale hematite (R4399) from Bayferrox, while the goethite standard mineral used is a pure yellow nanoscale goethite (SY610) from Hover Color.

The samples are first ground to less than 200 mesh using an agate mortar, placed on glass slides, diluted with distilled water to a slurry and spread evenly, dried at low temperature (< 40 °C), and spectroscopically scanned using a Lambd 900 UV/VIS/NIR spectrophotometer from Perkin-Elmer, with a measurement interval of 2 nm, the measured band is 400–700 nm, and the visible band is divided into six different color bands, violet = 400–450 nm, blue = 450–490 nm, green = 490–560 nm, yellow = 560–590 nm, orange = 590–630 nm, red = 630–700 nm.

Stepwise multiple linear regression analysis is performed on the added hematite content and reflectance of each colour band (Supplementary Table 2), resulting in a calibration equation: Hematite% = 8.88 - 0.329 × green - 0.055 × red. Sample pretreatment and measurements are carried out at the Ministry-of-Education Key Laboratory of Surficial Geochemistry, Nanjing University.

Supplementary Table 2. Percentage reflectance in standard color bands of calibration samples.

Sample	violet	blue	green	yellow	orange	red	Goethite (%)	Hematite (%)
BY 01	10.32	8.51	16.85	10.78	18.64	34.90	0.20	1.80
BY 02	9.95	8.45	17.19	10.97	18.69	34.76	0.40	1.60
BY 03	9.68	8.49	17.85	11.25	18.59	34.14	0.60	1.40
BY 04	9.46	8.48	18.29	11.44	18.54	33.78	0.80	1.20
BY 05	9.08	8.36	18.73	11.72	18.57	33.53	1.00	1.00
BY 06	8.92	8.35	19.34	11.97	18.47	32.96	1.20	0.80
BY 07	8.66	8.33	20.15	12.27	18.29	32.30	1.40	0.60
BY 08	8.36	8.23	20.85	12.60	18.18	31.77	1.60	0.40
BY 09	7.95	8.07	21.76	13.04	18.04	31.15	1.80	0.20
BY 10	12.92	12.04	24.34	11.02	14.59	25.10	0.10	0.00
BY 11	12.38	11.66	24.35	11.24	14.85	25.51	0.20	0.00
BY 12	11.33	10.98	24.32	11.65	15.37	26.34	0.40	0.00
BY 13	10.44	10.32	24.17	12.04	15.87	27.16	0.60	0.00
BY 14	9.81	9.80	23.98	12.34	16.26	27.81	0.80	0.00
BY 15	9.43	9.49	23.86	12.54	16.51	28.18	1.00	0.00
BY 16	8.55	8.71	23.40	13.01	17.13	29.20	1.50	0.00
BY 17	8.10	8.18	22.93	13.33	17.56	29.89	2.00	0.00
BY 18	13.55	12.09	23.38	10.81	14.70	25.47	0.00	0.10
BY 19	13.35	11.72	22.57	10.84	15.13	26.39	0.00	0.20
BY 20	12.96	11.07	21.24	10.86	15.86	28.01	0.00	0.40
BY 21	12.36	10.44	20.12	10.90	16.56	29.63	0.00	0.60
BY 22	11.91	9.95	19.25	10.90	17.10	30.89	0.00	0.80
BY 23	11.73	9.69	18.69	10.82	17.39	31.68	0.00	1.00
BY 24	11.10	9.00	17.31	10.65	18.14	33.79	0.00	1.50
BY 25	10.81	8.65	16.50	10.42	18.51	35.12	0.00	2.00
BY 26	13.81	12.60	24.39	10.70	14.14	24.37	0.00	0.00

4. **Results**

4.1. Error estimation

Supplementary Table 3. Estimated uncertainties for each parameter measured in this study.

**Parameter**	**Estimation method**	**Aliquots**	**Samples**	**Relative error**	**Standard deviation**	**Error**	**Variation in the Madang profile**
Mean grain size (Mz)	Replication analysis	24			0.4 μm		10.6-13.5 μm
Magnetic susceptibility (MS)	Replication analysis	20			$0.5\times10^{-8}m^3kg^{-1}$		$13\text{-}150\times10^{-8}m^3kg^{-1}$
DCB-extractable iron (FeD)	Replication analysis	21			0.02%		1.16-4.63%
Total iron (FeT)	Standard sample			<2%			4.14-6.38%
Hematite (Hm)	Known sample		7			0.03%	0.19-0.88%
Redness (a*)	Replication analysis	60			0.12		6.82-10.77

4.2. Grain size distribution

The representative particle size frequency distribution curves from each unit of the Madang profile are relatively consistent, and all show a morphologically asymmetric distribution, with silt as the major component, and none of them show a coarse tail.

The particle size frequency distribution curves of the Madang samples show that there exist three peaks with corresponding mode sizes of ~0.9 μm, ~7 μm and ~30 μm respectively. Each peak represents a component corresponding to a different dynamic process. The <1 μm particles may be the pedogenic component[3], the 1-10 μm particles are the distal component, and the 16-32 μm particles are the proximal component (Supplementary Fig. 4). Therefore, the Madang loess has typical characteristics of dust sediments, consistent with the typical loess of the Loess Plateau.

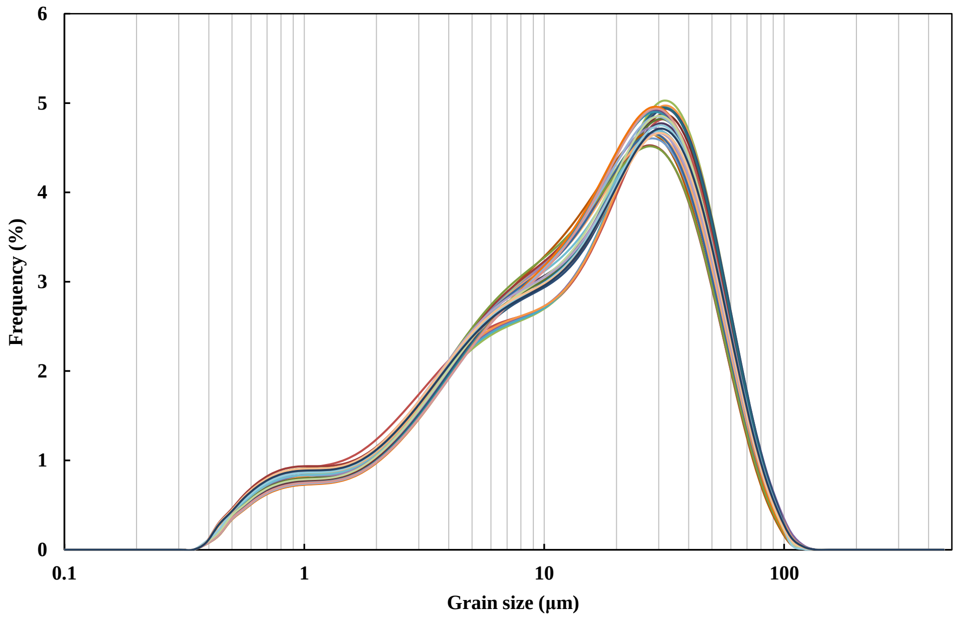

Supplementary Fig. 4. Grain size frequency curve of samples from different units of the Madang profile.

4.3. OSL ages

Supplementary Table 4. OSL dating results for quartz from the Madang profile.

Sample	Depth (cm)	Grain size (μm)	Water content (%)	U (ppm)	Th (ppm)	K (%)	De (Gy)	Dose rate (Gy/ka)	Age (ka)	Aliqout number
A8-40	40	4-11	17.1	3.20 ± 0.16	16.01 ± 0.80	1.73 ± 0.03	114.0 ± 2.3	3.79 ± 0.26	30.1 ± 2.2	6
A17-85	85	4-11	17.1	3.11 ± 0.16	15.65 ± 0.78	1.79 ± 0.04	149.6 ± 1.8	3.78 ± 0.26	39.6 ± 2.7	6
MD-P-0.6	155	4-11	13.2	3.06 ± 0.15	13.42 ± 0.67	1.75 ± 0.03	246.7 ± 4.2	3.69 ± 0.26	66.8 ± 4.8	6
MD-P-1.2	215	4-11	15.4	3.07 ± 0.15	13.66 ± 0.68	1.71 ± 0.03	262.6 ± 9.3	3.58 ± 0.25	73.3 ± 5.8	6
MD-P-1.5	245	4-11	17.4	3.21 ± 0.12	13.90 ± 0.38	1.71 ± 0.05	271.5 ± 5.2	3.56 ± 0.25	76.2 ± 5.5	6
MD-P-4.5	350	4-11	17.6	2.44 ± 0.10	15.40 ± 0.42	1.96 ± 0.06	312.8 ± 6.7	3.66 ± 0.25	85.5 ± 6.0	6

4.4. Time scale and proxy indicators

Supplementary Table 5. Tie points between the depth of the Madang profile and the age of the Luochuan profile[4].

Depth (cm)	100	260	445	755	860	945	1020	1320	1540
Age (ka)	56	78	128	186	195	233	242	281	334

Supplementary Table 6. Ages and proxy indicators for samples at different depths

Depth (cm)	Age (ka)	MS ($10^{-8}m^3kg^{-1}$)	FDS ($10^{-8}m^3kg^{-1}$)	FeD (%)	FeT (%)	FeD/FeT	Hm (%)	Mz (μm)	a*
5	42.9	29.2	3.3	1.60	4.42	0.36	0.23	12.9	6.82
10	43.6	27.9	3.2	1.82	4.51	0.40	0.27	13.1	
15	44.3	29.4	3.6	2.08	4.59	0.45	0.28	13.0	
20	45.0	25.8	2.7	1.34	4.93	0.27	0.34	12.5	8.18
25	45.7	33.6	4.5	2.24	5.19	0.43	0.43	12.3	
30	46.4	31.9	4.5	2.17	5.33	0.41	0.42	11.6	
35	47.1	33.1	3.1	2.76	5.32	0.52	0.43	11.3	
40	47.8	40.2	3.1	2.51	5.47	0.46	0.41	11.2	
45	48.4	40.7	4.4	2.78	5.80	0.48	0.44	10.7	
50	49.1	40.0	4.1	2.02	5.70	0.35	0.47	10.8	9.08
55	49.8	43.1	5.5	2.16	5.87	0.37	0.52	10.7	
60	50.5	38.2	4.1	2.26	5.95	0.38	0.53	11.0	
65	51.2	37.3	6.1	1.80	6.07	0.30	0.49	11.1	
70	51.9	48.3	5.8	2.34	6.15	0.38	0.56	11.7	
75	52.6	41.0	5.9	2.52	5.67	0.44	0.46	10.6	
80	53.3	39.9	5.2	2.49	5.61	0.44	0.41	11.1	8.35
85	53.9	45.0	4.5	2.77	5.75	0.48	0.44	11.1	
90	54.6	46.1	5.3	1.89	5.93	0.32	0.45	11.5	
95	55.3	39.3	4.6	2.13	6.10	0.35	0.45	11.5	
100	56.0	48.8	7.1	2.26	6.04	0.37	0.50	10.9	9.1
105	56.7	42.4	7.0	1.87	6.03	0.31	0.50	11.2	
110	57.4	36.1	7.0	1.86	4.96	0.38	0.30	11.1	
115	58.1	43.7	6.8	2.76	5.71	0.48	0.45	11.1	
120	58.8	40.2	6.3	2.97	5.89	0.51	0.44	11.5	
125	59.4	39.5	4.3	2.17	5.14	0.42	0.30	11.3	
130	60.1	37.8	6.6	2.35	5.28	0.44	0.35	11.0	8.12
135	60.8	42.4	6.1	2.54	5.43	0.47	0.31	10.6	

140	61.5	35.4	5.6	2.28	4.74	0.48	0.27	11.5	
145	62.2	35.8	4.7	1.92	4.60	0.42	0.23	11.3	
150	62.9	30.6	3.8	1.81	4.55	0.40	0.20	11.6	
155	63.6	37.4	3.8	1.82	4.51	0.40	0.23	12.0	
160	64.3	34.4	3.7	1.80	4.71	0.38	0.27	11.9	
165	64.9	38.5	5.1	1.71	4.79	0.36	0.28	11.4	
170	65.6	34.7	3.8	1.51	4.46	0.34	0.19	11.6	
175	66.3	32.0	4.0	1.91	4.54	0.42	0.22	12.0	
180	67.0	32.1	3.9	2.12	4.52	0.47	0.22	11.9	
185	67.7	34.7	4.2	1.81	4.45	0.41	0.23	12.0	
190	68.4	31.3	3.3	2.08	4.51	0.46	0.20	12.0	
195	69.1	31.6	3.4	2.17	4.46	0.49	0.22	11.4	7.19
200	69.8	34.0	3.6	1.98	4.27	0.46	0.24	12.2	
205	70.4	33.4	4.2	1.59	4.16	0.38	0.22	13.0	
210	71.1	34.3	4.0	2.02	4.20	0.48	0.25	12.8	
215	71.8	39.3	4.9	1.61	4.14	0.39	0.28	12.7	7.51
220	72.5	37.6	3.5	2.58	4.22	0.61	0.27	12.9	
225	73.2	36.9	2.6	2.13	4.17	0.51	0.24	12.9	7.33
230	73.9	37.1	2.9	1.91	4.25	0.45	0.23	12.9	
235	74.6	38.1	4.5	1.36	4.17	0.33	0.28	12.6	
240	75.3	37.8	4.3	2.42	4.18	0.58	0.23	12.8	
245	75.9	38.4	4.0	2.28	4.20	0.54	0.30	12.6	
250	76.6	37.1	3.0	2.36	4.25	0.56	0.29	12.3	
255	77.3	60.3	7.1	2.92	5.63	0.52	0.47	13.1	
260	78.0	117.4	13.6	2.44	5.97	0.41	0.60	13.0	8.96
265	79.4	83.5	9.9	2.86	5.63	0.51	0.59	12.9	9.57
270	80.7	88.0	10.1	2.75	5.67	0.48	0.63	13.0	
275	82.1	106.7	12.7	2.38	5.59	0.43	0.54	12.3	
280	83.4	85.8	9.9	2.98	5.80	0.51	0.50	12.9	
285	84.8	83.2	14.5	3.05	5.42	0.56	0.58	12.4	
290	86.1	109.3	14.2	2.46	5.38	0.46	0.57	12.9	9.27
295	87.5	99.2	10.9	2.67	5.67	0.47	0.51	13.0	
300	88.8	90.6	10.8	2.89	5.54	0.52	0.54	13.1	
305	90.2	111.9	10.1	2.89	5.52	0.52	0.57	13.5	
310	91.5	96.0	9.1	3.41	5.43	0.63	0.58	13.4	
315	92.9	78.3	8.3	3.25	5.75	0.56	0.54	13.0	8.6
320	94.2	100.3	12.9	3.07	5.63	0.55	0.55	12.4	9.15
325	95.6	92.5	12.5	2.91	5.97	0.49	0.56	12.8	

330	96.9	99.6	11.0	2.33	5.56	0.42	0.59	13.0	
335	98.3	106.3	10.2	2.61	5.57	0.47	0.60	12.9	
340	99.6	88.4	8.9	2.87	5.71	0.50	0.61	12.7	9.34
345	101.0	98.1	8.5	2.75	6.06	0.45	0.59	12.9	
350	102.3	99.4	10.1	3.03	5.59	0.54	0.59	13.2	
355	103.7	98.3	12.6	2.55	5.57	0.46	0.64	13.0	
360	105.0	99.5	9.2	2.73	5.69	0.48	0.58	12.5	
365	106.4	100.7	10.1	2.71	5.84	0.46	0.61	12.6	
370	107.7	86.4	8.3	2.67	5.66	0.47	0.63	13.3	
375	109.1	108.2	12.2	2.11	5.61	0.38	0.60	12.5	
380	110.4	99.2	12.8	1.67	5.63	0.30	0.55	13.2	
385	111.8	118.3	9.7	1.48	5.55	0.27	0.48	13.0	9.26
390	113.1	88.6	10.7	1.53	5.54	0.28	0.60	12.7	
395	114.5	102.2	13.2	1.21	5.54	0.22	0.63	13.0	
400	115.8	110.3	11.7	1.37	5.52	0.25	0.54	13.0	
405	117.2	97.4	11.3	1.20	5.50	0.22	0.72	13.3	9.46
410	118.5	97.1	9.4	1.97	5.82	0.34	0.64	13.2	
415	119.9	104.2	9.4	1.68	5.53	0.30	0.58	12.6	
420	121.2	113.4	10.4	2.05	5.55	0.37	0.63	12.5	
425	122.6	116.2	12.8	2.17	5.51	0.39	0.63	12.7	
430	123.9	119.3	9.7	2.59	5.51	0.47	0.63	13.0	9.36
435	125.3	112.3	10.1	2.31	5.54	0.42	0.62	12.8	
440	126.6	110.2	11.5	2.82	5.48	0.51	0.68	12.4	
445	128.0	118.3	11.5	2.39	5.48	0.44	0.70	12.7	
450	128.9	108.1	11.9	1.80	5.50	0.33	0.49	12.5	8.4
455	129.9	97.9	12.3	1.64	5.53	0.30	0.63	12.6	
460	130.8	99.5	10.6	1.90	5.54	0.34	0.70	11.8	
465	131.7	94.2	9.2	1.82	5.52	0.33	0.64	12.2	
470	132.7	87.6	9.0	1.81	5.52	0.33	0.64	12.1	9.36
475	133.6	76.5	8.0	1.50	5.62	0.27	0.58	12.1	
480	134.5	67.5	5.7	1.71	5.51	0.31	0.52	12.1	
485	135.5	62.1	5.2	1.44	5.47	0.26	0.56	12.1	
490	136.4	53.3	3.0	1.38	5.60	0.25	0.51	12.5	
495	137.4	40.3	3.8	2.20	5.53	0.40	0.51	12.0	
500	138.3	42.5	3.5	1.95	5.60	0.35	0.51	12.2	
505	139.2	40.7	1.2	2.05	5.52	0.37	0.51	12.0	
510	140.2	34.6	1.5	1.17	5.46	0.21	0.52	11.9	8.33
515	141.1	36.8	1.1	1.29	5.61	0.23	0.56	12.2	

520	142.0	37.1	1.8	2.64	5.51	0.48	0.49	11.9	8.25
525	143.0	36.5	2.2	2.07	5.51	0.38	0.52	12.2	
530	143.9	31.7	2.4	2.70	5.49	0.49	0.51	12.1	
535	144.8	32.1	0.8	2.42	5.50	0.44	0.49	12.0	8.28
540	145.8	34.5	1.7	2.60	5.56	0.47	0.51	11.8	
545	146.7	22.5	1.5	2.92	5.53	0.53	0.51	12.0	8.06
550	147.6	30.9	2.3	3.13	5.17	0.60	0.61	11.9	
555	148.6	20.7	2.1	2.44	5.46	0.45	0.56	11.6	
560	149.5	21.7	1.6	2.98	5.58	0.53	0.55	11.8	
565	150.5	31.9	1.4	2.74	5.51	0.50	0.49	11.9	8.26
570	151.4	27.0	2.5	1.35	5.44	0.25	0.52	11.8	
575	152.3	27.8	0.6	2.68	5.43	0.49	0.49	11.9	
580	153.3	24.1	2.3	1.58	5.43	0.29	0.49	11.7	
585	154.2	24.0	1.9	1.80	5.40	0.33	0.45	11.9	7.93
590	155.1	21.9	2.4	1.16	5.37	0.22	0.46	12.2	
595	156.1	20.4	1.3	1.37	5.36	0.26	0.47	11.6	
600	157.0	20.8	1.4	2.92	5.35	0.55	0.48	11.7	
605	157.9	20.6	0.9	2.74	5.40	0.51	0.47	11.7	8.1
610	158.9	16.9	1.7	2.60	5.35	0.49	0.46	11.9	
615	159.8	13.1	0.8	2.14	5.54	0.39	0.47	11.9	8.11
620	160.7	22.3	0.7	3.24	5.60	0.58	0.52	12.1	
625	161.7	14.1	0.8	3.61	5.52	0.65	0.54	12.2	
630	162.6	30.5	0.7	3.41	5.50	0.62	0.52	12.3	8.34
635	163.5	26.7	2.0	3.14	5.56	0.57	0.47	12.2	
640	164.5	28.8	1.7	2.70	5.41	0.50	0.47	12.4	
645	165.4	26.7	1.0	2.38	5.34	0.45	0.52	12.3	
650	166.4	24.8	1.4	2.39	5.31	0.45	0.52	12.4	8.72
655	167.3	27.3	0.6	2.35	5.32	0.44	0.56	12.6	
660	168.2	30.1	1.3	2.35	5.33	0.44	0.57	12.5	8.74
665	169.2	26.0	1.7	2.11	5.32	0.40	0.59	12.8	
670	170.1	24.3	1.4	1.75	5.29	0.33	0.56	12.6	
675	171.0	23.9	1.5	1.38	5.12	0.27	0.47	12.5	
680	172.0	25.1	1.1	1.60	4.96	0.32	0.55	12.9	
685	172.9	24.0	0.7	1.83	4.97	0.37	0.47	13.2	
690	173.8	25.0	1.6	2.12	5.07	0.42	0.55	12.7	
695	174.8	25.0	1.6	2.20	5.06	0.43	0.56	12.8	
700	175.7	21.0	1.4	2.00	5.11	0.39	0.54	12.4	
705	176.6	24.5	2.2	1.72	5.13	0.34	0.59	12.2	

710	177.6	24.6	1.9	1.55	5.17	0.30	0.60	11.9	
715	178.5	21.9	2.3	1.70	4.30	0.40	0.51	12.0	
720	179.5	21.9	2.3	2.16	5.31	0.41	0.60	11.7	
725	180.4	23.8	2.8	2.22	5.33	0.42	0.58	11.7	
730	181.3	21.7	2.4	2.09	5.26	0.40	0.59	11.9	
735	182.3	23.4	1.6	1.90	5.19	0.37	0.55	11.7	
740	183.2	21.4	2.4	1.23	5.10	0.24	0.58	11.9	8.8
745	184.1	21.0	2.1	1.65	5.15	0.32	0.54	12.1	
750	185.1	21.4	2.7	1.46	5.01	0.29	0.57	12.2	
755	186.0	19.0	2.6	1.48	4.92	0.30	0.59	12.5	
760	186.4	22.3	3.2	1.55	5.27	0.29	0.66	12.2	
765	186.9	26.9	3.9	1.28	5.23	0.24	0.67	12.6	9.42
770	187.3	37.8	4.1	1.88	5.48	0.34	0.72	11.7	10.09
775	187.7	44.4	6.6	2.17	5.66	0.38	0.77	11.7	
780	188.1	53.7	6.2	2.49	5.71	0.44	0.72	11.6	10.11
785	188.6	51.6	7.0	1.72	5.79	0.30	0.78	11.7	
790	189.0	66.0	7.7	2.30	5.86	0.39	0.74	11.4	
795	189.4	68.4	10.3	1.92	5.84	0.33	0.81	11.3	10.38
800	189.9	70.7	7.3	2.23	5.86	0.38	0.76	11.6	
805	190.3	73.0	10.3	2.33	5.91	0.39	0.78	12.2	
810	190.7	76.4	7.7	1.88	5.83	0.32	0.77	11.5	
815	191.1	75.8	7.8	1.95	5.78	0.34	0.81	11.6	
820	191.6	81.0	9.9	1.83	5.84	0.31	0.74	12.7	10.03
825	192.0	79.0	7.8	1.86	5.76	0.32	0.76	12.7	
830	192.4	79.0	10.1	2.43	5.74	0.42	0.73	12.1	
835	192.9	67.2	7.4	3.12	5.79	0.54	0.66	12.6	9.87
840	193.3	66.8	6.7	2.45	5.86	0.42	0.76	12.1	
845	193.7	68.2	10.0	2.74	5.71	0.48	0.73	11.9	
850	194.1	68.8	11.5	2.74	5.89	0.46	0.73	11.9	
855	194.6	88.2	8.6	2.72	5.75	0.47	0.74	11.9	
860	195.0	98.9	11.0	2.53	5.70	0.44	0.69	12.1	9.99
865	197.2	90.0	8.3	2.82	5.90	0.48	0.69	12.0	
870	199.5	82.3	9.6	2.55	5.61	0.45	0.69	12.5	
875	201.7	71.2	6.9	2.28	5.74	0.40	0.70	11.6	9.92
880	203.9	47.9	5.3	1.60	5.79	0.28	0.68	11.8	9.99
885	206.2	76.7	7.0	1.44	5.54	0.26	0.67	12.4	
890	208.4	72.4	7.5	2.13	5.56	0.38	0.48	12.6	9.74
895	210.6	50.3	6.3	3.14	6.17	0.51	0.70	12.5	

900	212.9	71.3	8.6	2.69	5.49	0.49	0.69	12.2	
905	215.1	41.9	3.4	2.91	5.52	0.53	0.56	11.9	9.02
910	217.4	35.0	3.6	2.41	5.36	0.45	0.56	11.7	8.8
915	219.6	38.8	3.0	2.66	5.29	0.50	0.54	11.4	8.96
920	221.8	30.2	3.0	1.95	5.58	0.35	0.55	11.4	
925	224.1	33.4	2.7	1.61	5.34	0.30	0.53	11.5	
930	226.3	34.7	3.7	2.13	5.39	0.39	0.59	11.1	
935	228.5	32.0	5.3	2.13	5.51	0.39	0.61	11.2	
940	230.8	26.6	4.3	1.58	5.32	0.30	0.64	11.1	
945	233.0	26.0	4.2	1.37	5.65	0.24	0.58	11.5	8.78
950	233.6	28.6	2.3	1.92	5.50	0.35	0.61	11.3	
955	234.2	27.9	3.0	1.79	5.35	0.33	0.59	11.3	
960	234.8	33.4	2.6	2.21	5.42	0.41	0.57	11.2	
965	235.4	42.1	4.9	1.82	5.14	0.35	0.54	11.7	
970	236.0	46.8	5.6	1.68	5.11	0.33	0.49	11.7	8.63
975	236.6	49.6	4.1	1.80	5.02	0.36	0.47	11.7	
980	237.2	48.0	4.3	2.90	5.04	0.58	0.50	11.7	
985	237.8	49.3	4.8	2.37	4.89	0.49	0.49	12.1	8.68
990	238.4	42.3	3.9	2.10	4.96	0.42	0.49	13.1	
995	239.0	63.1	7.4	1.87	5.62	0.33	0.63	12.6	9.35
1000	239.6	48.8	5.2	3.04	5.33	0.57	0.56	12.5	
1005	240.2	51.5	5.8	1.51	5.75	0.26	0.60	11.9	9.05
1010	240.8	76.4	7.6	3.13	5.95	0.53	0.62	12.8	
1015	241.4	71.7	10.8	1.66	5.90	0.28	0.61	12.0	
1020	242.0	88.9	9.7	1.65	5.94	0.28	0.63	12.4	9.48
1025	242.7	68.9	8.7	1.86	5.93	0.31	0.61	12.3	9.43
1030	243.3	71.7	6.6	1.62	5.91	0.27	0.60	12.5	
1035	244.0	60.0	6.5	1.75	5.94	0.30	0.60	12.3	
1040	244.6	49.9	4.1	2.89	5.97	0.48	0.55	12.5	9.11
1045	245.3	52.6	4.9	1.50	5.96	0.25	0.59	13.3	
1050	245.9	46.3	3.8	4.27	5.80	0.74	0.53	12.2	
1055	246.6	36.2	3.9	4.04	5.86	0.69	0.52	12.0	
1060	247.2	38.9	3.0	4.25	6.05	0.70	0.51	12.5	
1065	247.9	30.8	3.2	4.63	6.17	0.75	0.50	12.8	
1070	248.5	29.9	2.8	3.26	5.90	0.55	0.37	13.2	
1075	249.2	30.2	1.8	4.24	5.88	0.72	0.44	12.8	8.4
1080	249.8	31.9	1.7	3.56	5.92	0.60	0.44	12.3	
1085	250.5	29.9	1.2	3.13	6.07	0.52	0.48	12.7	

1090	251.1	36.5	2.5	3.05	5.91	0.52	0.51	13.1	
1095	251.8	40.0	1.9	2.55	5.93	0.43	0.47	13.0	8.27
1100	252.4	26.1	1.7	2.50	6.03	0.41	0.49	12.6	8.7
1105	253.1	32.8	1.2	1.95	5.55	0.35	0.48	13.0	
1110	253.7	25.4	2.2	2.20	5.65	0.39	0.48	13.5	
1115	254.4	37.0	1.5	2.42	5.76	0.42	0.45	13.0	
1120	255.0	29.5	2.3	2.60	5.87	0.44	0.48	12.6	
1125	255.7	34.8	1.2	4.04	6.38	0.63	0.48	13.3	
1130	256.3	38.8	2.2	3.58	5.54	0.65	0.49	13.0	8.76
1135	257.0	30.0	2.0	3.17	5.52	0.58	0.50	13.3	
1140	257.6	30.0	1.9	3.34	5.53	0.60	0.41	13.1	
1145	258.3	38.3	1.9	3.48	5.78	0.60	0.46	13.4	
1150	258.9	38.2	1.5	2.85	5.50	0.52	0.48	13.0	
1155	259.6	35.5	2.3	3.20	5.53	0.58	0.47	12.8	
1160	260.2	33.8	1.6	3.15	5.31	0.59	0.48	13.2	
1165	260.9	32.7	0.7	2.86	5.37	0.53	0.42	13.0	7.98
1170	261.5	35.8	1.1	3.15	5.36	0.59	0.42	13.0	
1175	262.2	36.8	0.8	3.37	5.43	0.62	0.43	13.1	
1180	262.8	29.5	0.8	2.73	5.77	0.47	0.42	13.0	
1185	263.5	31.5	1.1	2.98	5.45	0.55	0.47	13.3	
1190	264.1	34.1	1.9	2.94	5.36	0.55	0.50	13.0	
1195	264.8	35.8	1.0	3.46	5.48	0.63	0.49	12.9	8.46
1200	265.4	33.8	2.0	3.12	5.40	0.58	0.50	12.9	
1205	266.1	32.4	1.0	2.72	5.33	0.51	0.50	12.8	
1210	266.7	33.3	1.5	3.33	5.38	0.62	0.48	13.0	
1215	267.4	34.4	0.9	3.66	5.37	0.68	0.47	13.1	
1220	268.0	33.5	1.6	2.89	5.38	0.54	0.47	13.0	
1225	268.7	33.7	1.7	2.57	5.38	0.48	0.44	12.8	
1230	269.3	32.8	1.4	2.43	5.41	0.45	0.45	12.9	
1235	270.0	29.8	1.1	3.12	5.35	0.58	0.49	13.3	8.61
1240	270.6	33.0	2.1	2.73	5.29	0.52	0.46	13.3	
1245	271.3	33.0	1.6	2.99	5.36	0.56	0.45	13.1	
1250	271.9	33.0	1.4	2.89	5.32	0.54	0.45	13.1	
1255	272.6	29.0	1.7	2.74	5.35	0.51	0.42	13.0	
1260	273.2	31.7	0.9	3.51	5.36	0.66	0.47	13.5	
1265	273.9	32.5	1.6	3.34	5.37	0.62	0.47	13.5	8.38
1270	274.5	30.5	1.4	2.59	5.23	0.50	0.41	13.2	
1275	275.1	32.9	2.2	1.94	5.34	0.36	0.44	13.5	

1280	275.8	31.1	1.1	1.69	5.32	0.32	0.47	12.9	
1285	276.4	31.4	1.0	1.97	5.31	0.37	0.43	13.4	
1290	277.1	28.9	1.4	1.54	5.37	0.29	0.46	13.0	8.35
1295	277.7	32.9	2.1	2.39	5.32	0.45	0.47	13.0	
1300	278.4	33.3	1.0	3.08	5.31	0.58	0.46	13.0	
1305	279.0	28.7	1.2	2.93	5.24	0.56	0.44	12.9	
1310	279.7	32.6	1.1	3.38	5.35	0.63	0.47	13.2	
1315	280.3	30.0	1.1	3.21	5.24	0.61	0.44	13.2	
1320	281.0	32.9	1.5	2.92	5.27	0.55	0.47	12.9	
1330	283.4	131.9	15.9	3.66	5.72	0.64	0.83	11.4	10.34
1340	285.8	142.8	16.4	3.58	5.74	0.62	0.84	11.9	10.65
1350	288.2	130.9	15.1	3.75	5.74	0.65	0.88	11.9	10.71
1360	290.6	125.8	16.2	3.03	5.53	0.55	0.81	12.2	10.38
1370	293.0	131.8	16.1	3.65	5.71	0.64	0.83	12.1	10.77
1380	295.5	127.9	14.7	2.55	5.67	0.45	0.81	12.8	10.55
1390	297.9	141.7	16.9	2.72	5.62	0.48	0.81	12.6	10.77
1400	300.3	137.9	16.8	3.51	5.57	0.63	0.79	12.6	
1410	302.7	100.2	11.5	2.94	5.34	0.55	0.68	11.9	9.7
1420	305.1	104.9	13.1	2.32	5.38	0.43	0.71	12.2	
1430	307.5	106.3	13.3	2.92	5.42	0.54	0.72	11.9	
1440	309.9	114.6	15.3	2.30	5.41	0.43	0.76	12.2	10.15
1450	312.3	145.7	18.5	2.59	5.53	0.47	0.82	12.4	10.68
1460	314.7	150.2	7.1	2.82	5.49	0.51	0.83	12.9	10.75
1470	317.1	135.0	16.3	2.37	5.49	0.43	0.79	12.8	10.62
1480	319.5	143.7	17.6	2.54	5.44	0.47	0.82	13.2	10.77
1490	322.0	123.9	16.6	3.06	5.43	0.56	0.77	12.9	10.46
1500	324.4	144.1	14.8	2.86	5.50	0.52	0.81	12.8	10.48
1510	326.8	125.2	14.4	3.20	5.48	0.59	0.76	12.5	10.36
1520	329.2	129.9	14.6	3.28	5.36	0.61	0.75	12.5	
1530	331.6	124.3	15.6	2.86	5.38	0.53	0.76	12.7	
1540	334.0	125.6	13.7	3.09	5.36	0.58	0.74	12.7	
1550	336.4	34.4	4.3	2.68	5.00	0.54	0.57	11.8	8.81
1560	338.8	27.7	2.7	2.42	5.02	0.48	0.59	11.8	
1570	341.2	30.9	3.4	3.04	4.96	0.61	0.53	11.7	
1580	343.6	26.8	4.8	2.31	5.05	0.46	0.55	11.3	8.65
1590	346.0	28.2	3.2	1.75	4.95	0.35	0.52	11.8	

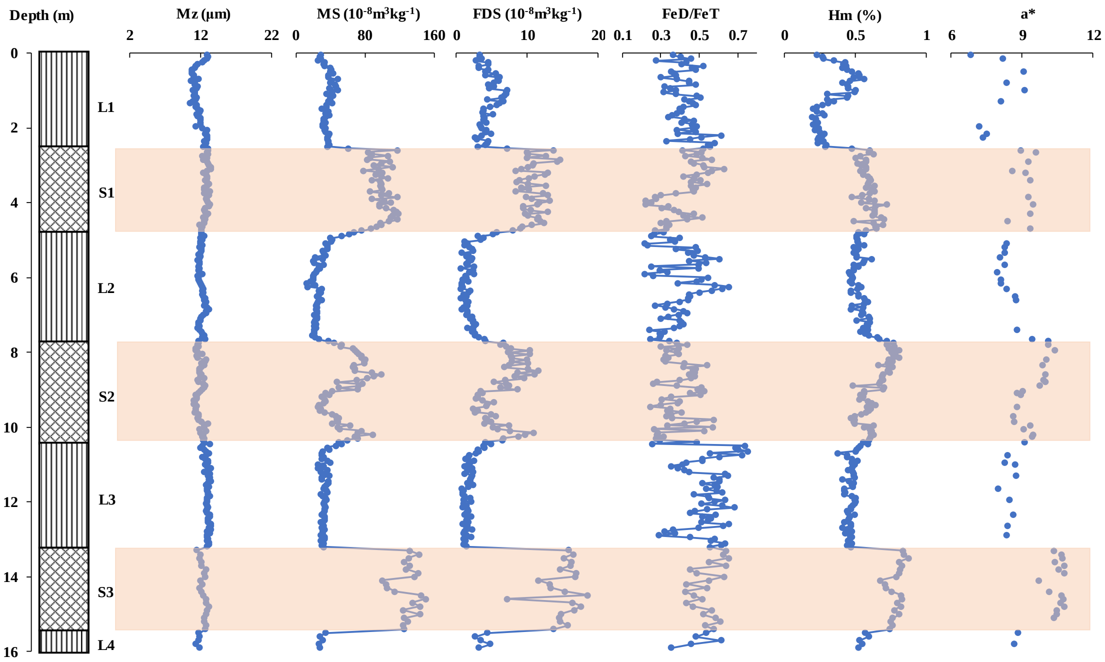

Supplementary Fig. 5. Down-profile variation of proxy indicators. The band in light orange represents the paleosol units. The paleosol units generally have relatively high values for magnetic susceptibility (MS), frequency-dependent magnetic susceptibility (FDS), hematite content (Hm) and redness (a*). This trend is not observed in the curves of mean grain size (Mz) and the ratio of DCB-extractable iron to total iron (FeD/FeT).

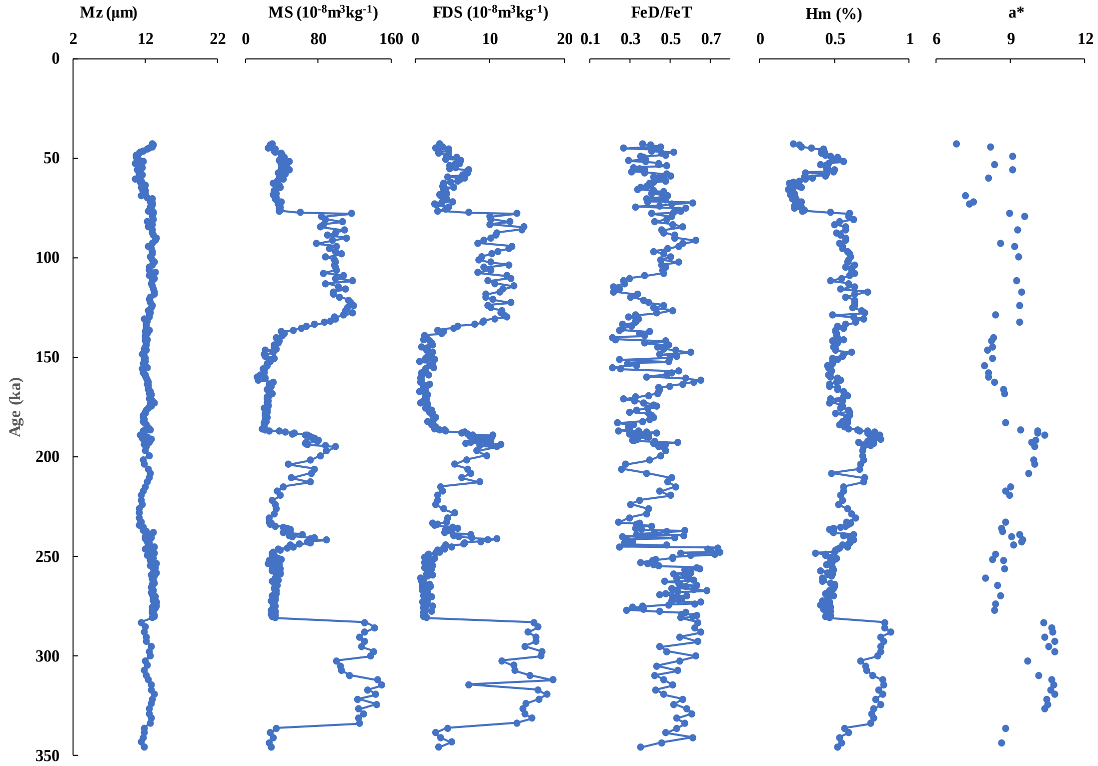

Supplementary Fig. 6. Temporal sequence of proxy indicators of the Madang profile. The sequences of magnetic susceptibility (MS), frequency-dependent magnetic susceptibility (FDS), hematite (Hm) content and redness (a*) show a ~100-kyr cycle. The sequence of DCB-extractable iron (FeD) shows a quasi-20-kyr cycle. The mean grain size (Mz) sequence shows no cycle.

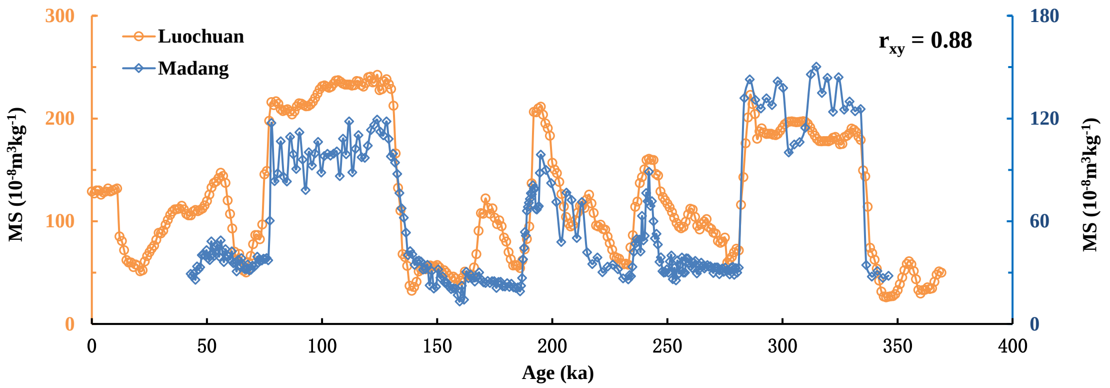

Supplementary Fig. 7. Comparison of magnetic susceptibility (MS) curves between Madang and Luochuan. Strong positive correlation (r_{xy} = 0.88) validates the reliability of the Madang timescale established with nine tie points.

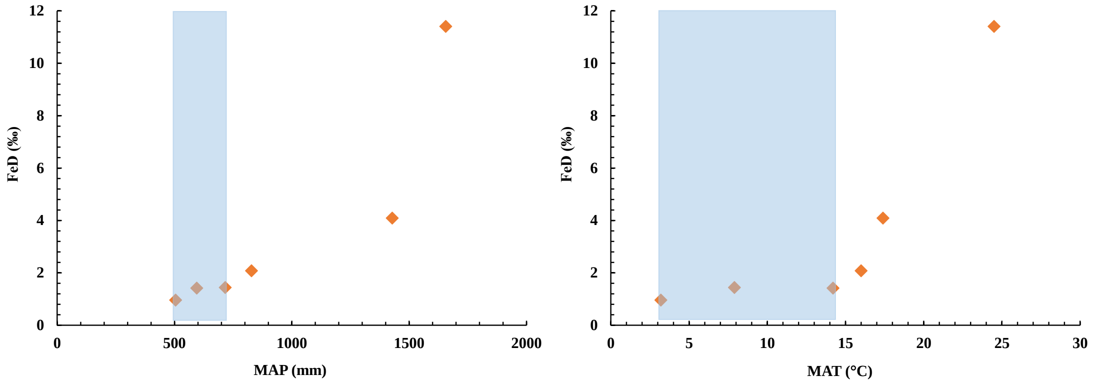

Supplementary Fig. 8. Dependence of DCB-extractable iron (FeD) in modern soil on the mean annual precipitation (MAP) and mean annual temperature (MAT)[5]. The increase in FeD is insignificant when MAP is below ~800 mm or MAT is below ~15 °C (marked with light blue band).

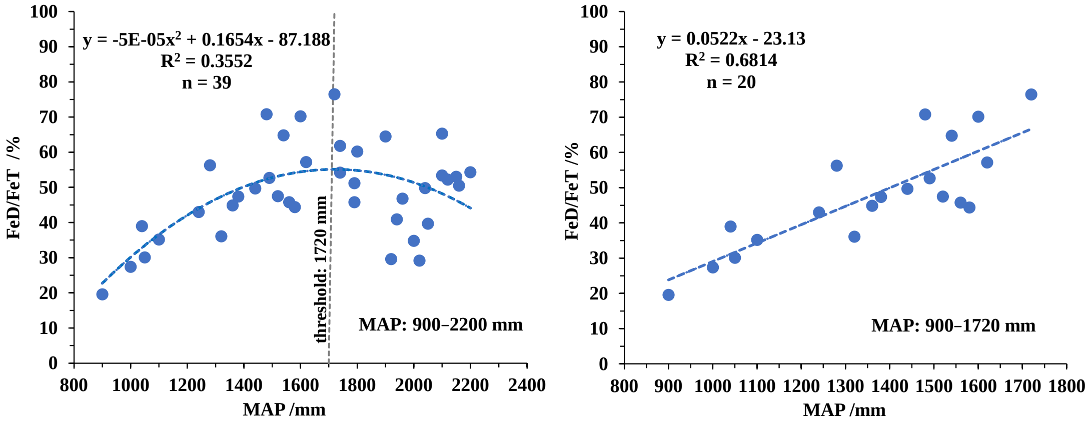

Supplementary Fig. 9. Dependence of the ratio of DCB-extractable iron (FeD) to total iron (FeT) in modern soil on mean annual precipitation (MAP)[6]. The soil samples were collected from Hainan Island, South China. The FeD/FeT generally increases with increasing MAP up to approximately 1720 mm, but decreases with further increases in MAP (left). A linear correlation is observed for MAP values below 1720 mm (right).

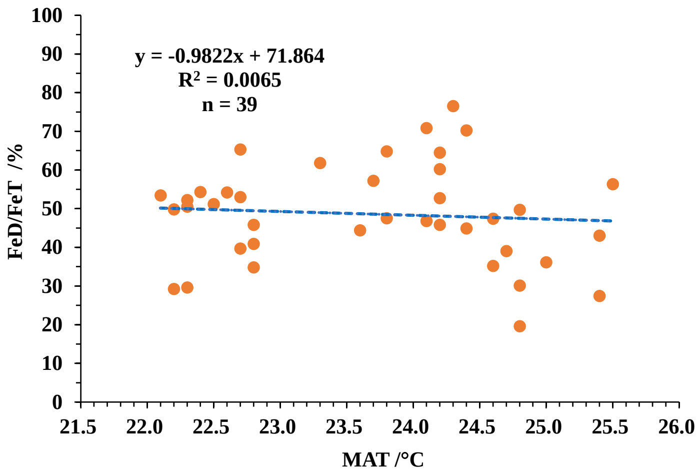

Supplementary Fig. 10. Relationship between the ratio of DCB-extractable iron (FeD) to total iron (FeT) in modern soil and mean annual temperature (MAT)[6]. The soil samples were collected from Hainan Island, South China. The data suggest that there is no significant correlation between FeD/FeT ratio and MAT.

Supplementary references

1. Jarvis, A., Reuter, H. I., Nelson, A. & Guevara, E. Hole-filled SRTM for the globe Version 4. Available from the CGIAR-CSI SRTM 90m Database http://srtm.csi.cgiar.org (2008).
2. Ji, J. et al. Rapid and quantitative measurement of hematite and goethite in the Chinese loess-paleosol sequence by diffuse reflectance spectroscopy. *Clays Clay Miner*. **50**, 208–216 (2002).
3. Sun, D. et al. The ultrafine component in Chinese loess and its variation over the past 7.6 Ma: implications for the history of pedogenesis. *Sedimentology* **58**, 916–935 (2011).
4. Lu, H. et al. Astronomical calibration of loess–paleosol deposits at Luochuan, central Chinese Loess Plateau, *Palaeogeogr*. *Palaeoclimatol*. *Palaeoecol.* **154**, 237–246 (1999).
5. Peng, J. et al. Investigating intra-aggregate microstructure characteristics and influencing factors of six soil types along a climatic gradient. *Catena* **210**, 105867 (2022).
6. Long, X. et al. Climatic thresholds for pedogenic iron oxides under aerobic conditions: Processes and their significance in paleoclimate reconstruction. *Quat. Sci. Rev.* **150**, 264–277 (2016).
